# De-institutionalisation and trans-institutionalisation - changing trends of inpatient care in Norwegian mental health institutions 1950-2007

**DOI:** 10.1186/1752-4458-3-28

**Published:** 2009-12-25

**Authors:** Per Bernhard Pedersen, Arnulf Kolstad

**Affiliations:** 1 SINTEF Technology and Society, Health Services Research, 7465 Trondheim, Norway; 2 Department of Psychology, Norwegian University of Science and Technology (NTNU), 7491 Trondheim, Norway

## Abstract

**Background:**

Over the last decades mental health services in most industrialised countries have been characterised by de-institutionalisation and different kinds of redistribution of patients. This article will examine the historical trends in Norway over the period 1950-2007, identify the patterns of change in service settings and discuss why the mental health services have been dramatically transformed in less than sixty years.

**Methods:**

The presentation of the trends in the Norwegian mental health services and the outline of the major changes in the patterns of inpatient care over the period 1950-2007 is founded on five indicators: The average inpatient population, the number of discharges during a year, the average length of stay, the number of beds or places, and the occupancy rate (average inpatient population/beds). Data are reported by institutional setting. Multiple sources of data are used. In some cases it has been necessary to interpolate data due to missing data.

**Results:**

New categories of institutions were established and closed during the 57 years period. De-hospitalisation started in Norway in the early 1970s, de-institutionalisation in general 15 years later. Six distinct periods are identified: The asylum period (-1955), institutionalisation and trans-institutionalisation (1955-65), stabilisation and onset of de-hospitalisation (1965-75), de-hospitalisation (1975-87), from nursing homes to community-based services (1988-98), and the national mental health program (1999-2007). There has been a significant reduction in the number of beds and in the average in-patient population. The average length of stay in institutions has been continuously reduced since 1955. The number of patients actually treated in psychiatric institutions has increased significantly. Accessibility, quality of care and treatment for most patients has improved during the period. The mental health system in Norway has recently been evaluated as better than the systems in USA, England and Canada.

**Conclusions:**

De-institutionalisation means fewer *beds *but not fewer *patients *treated, neither in institutions in general nor in psychiatric hospitals. The periods represent different kinds of de-, trans-, and even re-institutionalisation. Expansion of the welfare state, increased professional focus on active treatment and increased focus on patients' preferences are the factors that best explain de-institutionalisation in Norway.

## Background

Over the last decades mental health services in most industrialised countries have been characterised by de-institutionalisation and different kinds of redistribution of patients. The downsizing of the large psychiatric institutions, especially the psychiatric hospitals, took place in steps, and the national reforms varied in their pace, fashion and in their consequences. This article will examine the historical trends in Norway, identify the patterns of change in service settings and discuss why the mental health services have been dramatically transformed in less than sixty years. The consequences for patients as well as for the health services providers are also commented upon.

The downsizing of the psychiatric hospitals started in the US and the UK in the mid-1950s. In most Western European countries, however, these developments first gained momentum during the 1970s. Since then, the universal slogans have been: 'de-institutionalisation' 'community-based', 'open' and 'decentralised' mental health services. The large asylums from the 19^th ^century have been closed or downsized, and the total number of beds in psychiatric hospitals has fallen dramatically [1-20]. Mental health services have been established in the community, albeit with significant variation between countries [13,21,22].

De-institutionalisation, meaning the contraction of traditional institutional settings [2] and especially a decline in the number of beds, is a process lasting for some decades. Fundamentally, de-institutionalization comprises three processes [18]: 1) the shift away from dependence on psychiatric hospitals; 2) 'trans-institutionalization' or an increase in the number of mental health beds in general hospitals (or other settings); 3) the growth of community-based inpatient and outpatient services for people with mental health disorders and problems. Although the processes and the pattern of change vary between countries [23], there has been a common direction of development. The reduction in the number of beds, especially in the large psychiatric hospitals (PHs), has in most countries been followed by an increase in the number of patients treated at outpatient clinics and in decentralized centres, in smaller psychiatric wards at general hospitals (GHDs), or in their own home. The large institutions are no longer used as a permanent home for psychiatric patients.

The services in Norway have gone through the same stages as in most other Western countries [24]: from high thresholds for being admitted as well as for being discharged, to more easy access to the services; from large scale de-institutionalization to community care; from long length of stay, to very short; from custodial care to active treatment. In this article we empirically trace the patterns of de-institutionalisation in Norway, focusing in detail on the different stages of the process, and the redistribution of psychiatric patients in the period 1950 to 2007. We address whether the process has been uniform and whether it is just trans-institutionalization that appears as de-institutionalization.

The period 1950 to 2007 is characterised by different stages of patient redistribution. In the 1950s, the number of inpatients increased in most psychiatric institutions in Norway, followed by periods of redistribution or trans-institutionalisation the following decades. Some kinds of treatment and care facilities have been closed down, while new ones have been established. Private care and psychiatric nursing homes (PNHs) have been abolished totally, while the inpatient population at psychiatric hospitals (PHs) has been considerably reduced. The number of beds in general hospitals and in smaller, decentralised institutions (the District psychiatric centres (DPCs)) has increased.

The institutions have also become smaller. In 1955, there were approximately 135-140 psychiatric institutions and GHDs, with an average inpatient population of approximately 75-80 patients. Seventeen institutions had more than 200 patients, the largest having 876. By 2007 there were 106 institutions with an average in-patient population of 38. Only ten institutions had more than 100 patients, the largest having 203. This development has been to the benefit of patients and staff.

The aims of this study are 1) To analyse the development of the mental health services in Norway over the last 50 years, with particular attention on institutional care. 2) To discuss different causes of the de-institutionalisation, and 3) Give a brief account of the consequences of the changes in the services when it comes to quality and accessibility of services, clinical outcome and for health policy. More specifically we want to focus on a) how is the relationship between de-, trans- and re-institutionalisation in the mental health services over the last 50 years, b) how to explain the transformation of the mental health services, especially the de-, trans- and re-institutionalisation over the period, c) what are the consequences of these processes for patients, mental health services and policy, d) the relationship between changes in the number of beds and the number of patients treated.

Most studies in this field usually focus on the decline of the psychiatric hospitals alone. There are few studies, if any, covering de-, trans- and re-institutionalisation for a period of more than 50 years, and covering the whole spectre of institutional care.

### The reasons for de-institutionalization

The reasons for the downsizing of the traditional institutional settings are many and varied, being associated with sociological, financial, pharmacological, administrative and legal changes [1,3,5,6,8,10,12,13,15-17,19-21,24,25] and include

• *The pharmacological revolution*. New drugs introduced in the early 1950s made it possible to keep patients outside the institutions [20,26].

• *The critique of psychiatry and the total institutions*. Both inside and outside the profession the ill-effects of prolonged stay within the large institutions were put forward from the 1950s [27-30].

• *The welfare state*. The public welfare services (disability pensions, public housing, etc.) made it possible for long term patients to live outside the large institutions [10].

• *Cost containment and the fiscal crisis of the state*. The de-institutionalisation of services followed in the wake of the 1973 oil crisis with increased pressures on public finances and might therefore be considered as a means of cost reduction, or at least cost containment.

• *A shift in the focus of services*. Increased professional focus on active and acute treatment and less emphasis on long-term care of the chronically ill [3].

• *Increased emphasis on patients' rights and preferences*

In Norway, most health services are owned and run by public authorities. Over the period studied most specialised services have been the responsibility of the 19 county councils. From 2002 this responsibility was transferred to the central government. The 430 local councils are responsible for providing primary care services.

## Materials and methods

To grasp when, and to what extent de-institutionalization has taken place in Norway, it is important to trace the timing of changes, using indicators that can be empirically validated.

The presentation of the trends in the Norwegian mental health services and the outline of the major changes in the patterns of inpatient care over the period 1950-2007 is founded on five indicators:

• The average inpatient population on a given day during the year (in-patient days/365)

• The number of discharges during a year (that is, administrative episodes, one patient may have more than one discharge)

• The average length of stay

• The number of beds or places

• The occupancy rate (average inpatient population/beds).

These data are used to identify distinct periods in the changes of services and make the basis for the discussion of how and why the redistribution of patients took place.

### Sources of data

Multiple sources of data are used. Statistics Norway has published data on beds in institutions and the inpatient population from before 1950. SINTEF provides data regarding the mental health services from 1991. For the period 1950-1979, data were obtained from the Statistics Norway's annual reports. From 1980 onwards these were supplemented by data files covering the activity at each institution. From 1998 data on inpatients are collected by SINTEF and The Norwegian Patient Register. Additional information has been collected from white papers and reports published by the Ministry of Health and Care Services and the Norwegian Directorate of Health. For some years it has been necessary to interpolate data due to missing data for groups of, or individual institutions.

Data will be displayed by type of institutional setting. The following categories are used:

• Mental Hospitals (MPs)

• Psychiatric departments in general hospitals (GHDs) and independent Psychiatric Clinics (PCs)

• District Psychiatric Centres (DPCs)

• Other institutions, mainly known as colonies for the insane until 1961, then renamed 'Psychiatric Nursing Homes' (PNHs)

• Private care

The PCs were independent psychiatric institutions, providing services similar to the GHDs. That is, active, short term inpatient treatment to people with less severe psychiatric diagnosis (neuroses etc.) or more severe diagnosis but with good prognosis. The PCs were usually operated by private, charitable organisations (or, in one case, by a university). They were, however, mainly financed through public reimbursements.

From 1990 onwards, psychiatric hospitals were often reorganised as departments of a general hospital and became administratively similar to the already existing psychiatric departments (GHDs). From 1991 and onwards the PHs, GHDs and the PCs are therefore reported as one category.

Data covers most institutions run by the (specialised) mental health services for adults. A few specialised institutions had to be excluded due to insufficient data. These institutions peaked in 1965 with 224 beds (1.4 percent of all beds in the services that year), and do not influence the general pattern of changes over the period. Institutions for children and adolescents have been excluded, partly because data are insufficient. In addition, services to these groups have for the most part been delivered on an out-patient basis. The number of beds never exceeded 300-400.

The indicators are presented as absolute numbers. During the 60 years period the Norwegian population has increased from 3.3 million in 1950 to 4.7 million in 2007, a growth of 44 percent. This will over time influence the rates. In a separate section at the end of the manuscript, we have included three tables that give population based ratios for beds, average inpatient population and discharges by year and institutional setting.

## Results

### Average inpatient population

**Figure 1:**
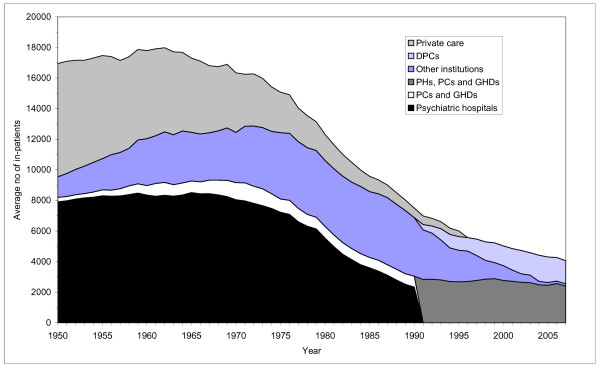
**Average number of inpatients by institutional setting**. Norwegian mental health institutions for adults. 1950-2007.

The average (in-patients day/365) absolute number and relative distribution of patients by service setting in the 1950 - 2007 period is illustrated in Figure 1.

From 1950 and up to the mid-1970s a considerable, but declining share of the psychiatric patients was in private care. The reduction of this service had a major impact on the subsequent development and patterns of institutionalised care. When this service was gradually closed down many patients were admitted to institutions. Data for this group is therefore included.

The average number of inpatients, including patients in private care, increased slightly from 1950 until the beginning of the 1960s. Due to increased population size, the ratio was, however, slightly reduced. Excluding patients in private care, however, the average inpatient population increased by 26 percent from in 1950 to 1964. The growth continued on a slower pace until it peaked in 1972. Since then, the average number of inpatients has been reduced by 69 percent.

### Discharges

**Figure 2:**
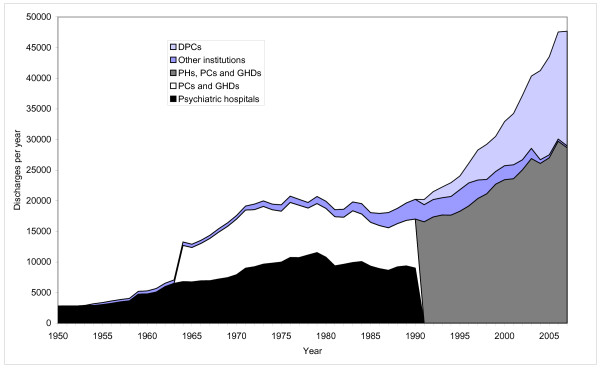
**Discharges from Norwegian mental health institutions for adults**. 1950-2007.

Figure 2 displays changes in the number of discharges over the period. For the PNH (other institutions) we lack data on discharges prior to 1954, and for GHDs and PCs we lack data prior to 1964.

The number of discharges from the PHs was multiplied by four between 1954 and 1979. The total number of discharges increased by 55 percent from 1964 to 1979. The number of discharges was reduced by thirteen percent over the next seven years. Despite low average number of inpatients, the PCs and GHDs were responsible for 40-50 percent of the discharges. Since 1986, the total number of discharged patients has increased by more than 150 percent.

### Average length of stay

**Figure 3:**
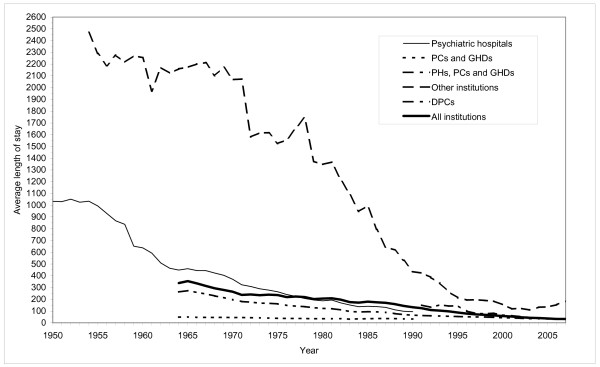
**Average length of stay in Norwegian mental health institutions for adults**. 1950-2007.

**Figure 4:**
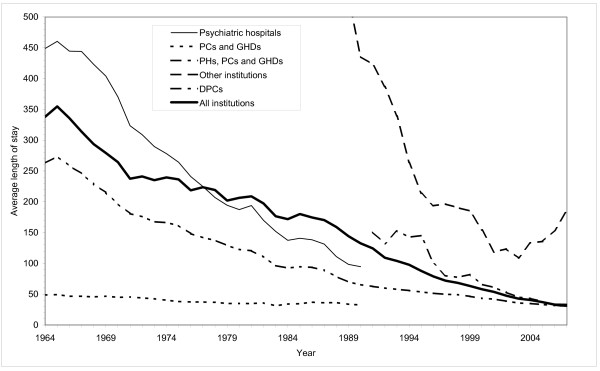
**Average length of stay in Norwegian mental health institutions for adults**. 1964-2007.

The increase in the number of discharges has only been possible through a drastic decrease in the average length of stay. The decrease is displayed in figure 3 and 4.

In the PHs the average length of stay was stable from 1950 to 1954, approximately 3 years. During the next decade it was reduced by more than 50 percent to 449 days in 1964, and has since decreased at a steady rate. By 1990, the average length of stay was 95 days, a reduction by more than 90 percent compared to 1950-54.

In 'other institutions' (PNH etc.), the average length of stay was fairly stable until 1971, only being reduced from 6 years in 1954 to 5 1/2 years in 1971. The PNHs were the home for many patients, not a place for psychiatric treatment. The patients were taken care of, given medical support and kept. These institutions eased the situations for the relatives and looked after people who were in need of physical and mental custody. Since the early 1970s the average length of stay has been drastically reduced, to an average of 109 days in 2003. By then, however, most of the PNHs had been closed down.

In the GHDs and the PCs the average length of stay was reduced from 49 days in 1964 to 33 days in 1990. For PHs, PCs and GHDs in total, the average length of stay was continuously reduced from 263 days in 1964 to 30 days in 2007. For the DPCs, introduced in the 1980s, average length of stay was reduced from 151 days in 1991 to 29 days in 2007. For mental health institutions in total, the average length of stay has been continuously reduced from 355 days in 1965 to 33 days in 2007, that is, from one year to one month on average.

### Beds and occupancy rate

**Figure 5:**
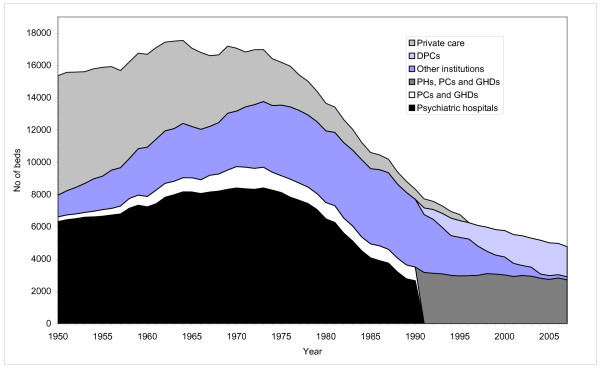
**Beds in Norwegian mental health institutions for adults and in private care**. 1950-2007.

**Figure 6:**
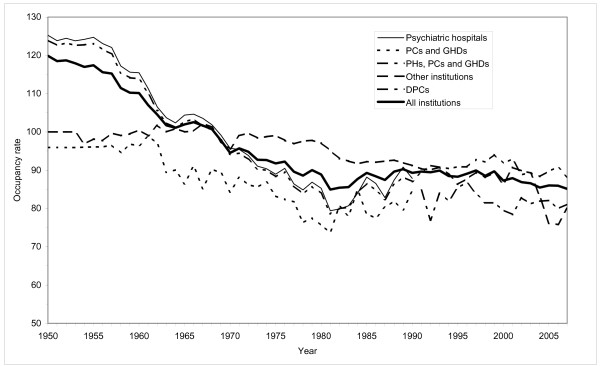
**Occupancy rate by institutional setting in Norwegian mental health institutions for adults**. 1950-2007.

Data on the number of beds by institutional setting is given in figure 5. Figure 6 gives the occupancy rate (average inpatients/beds).

Changes in the number of beds display the same general pattern as changes in the average number of inpatients. There are some differences, though. During the early 1950s, the mental hospitals were severely overcrowded with an occupancy rate of 125 percent. Throughout the whole period, the occupancy rate at the PHs and for institutions in general, has been reduced. As a consequence, there was as steeper increase in the number of beds than in the numbers of inpatients during the 1950s and 1960s. The total number of beds increased by 73 percent from 1950 to 1973. It has since been reduced by 65 percent.

### The six periods

Based on the data, the state of affairs in the mental health services can be separated into six distinct periods up to 2007:

• Before 1955: The asylum period

• 1955-65: Institutionalisation and trans-institutionalisation

• 1965-75: Inpatient population stabilized and the onset of de-hospitalization

• 1975-87: De-hospitalization

• 1988-96: From nursing homes to community based services• 1998-2008: The National Mental Health Programme.

These periods coincide with important policy shift, both in Norway and in other countries.

### Prior to 1955: From establishing asylums to overcrowding

The first purpose-built state psychiatric hospital in Norway, Gaustad asylum in Oslo, opened in 1855 with 270 beds. By 1921 Norway had 21 psychiatric asylums with 3972 beds. There were hardly any other facilities established at that time.

Due to the shortage of beds in psychiatric institutions, many patients were placed in private care during the first half of the 20^th ^century. Private providers/families received patients in their own home, usually on a farm. The patients took part in the running of the farm or in domestic activities. In addition, the farmer received payment, from the psychiatric services, from poor relief services or paid for by the patients themselves. The living conditions and welfare of the patients was to be under the supervision of an MD, usually the district medical officer. In some cases, the private care and supervision was also organised by PHs. By 1950 more than 7000 patients received this form of care.

The mental health services up to 1950s therefore consisted primarily of psychiatric hospitals (asylums) and private care. As illustrated in figure 1 and 5, the two caring forms still dominated the mental health services up to the mid-1950s. Some few 'nerve clinics' for the treatment of milder cases, especially patients diagnosed with 'neurosis' had been established by private charitable, humanistic and religious organizations, but they did not have any impact on the inconsolable situation in psychiatric hospitals.

The focus in the mental health services in this period was on long-term care, custody more than psychotherapy. Medical treatment, for instance shock therapy and lobotomy, was heavily used in the 1940s and 1950s. Between 1941 and 1956, 2500 patients were lobotomized at mental hospitals in Norway [31].

Overcrowded hospitals had been a recurrent problem since 1880 and reached its climax at the beginning of the 1950s. High pressure on the PHs with 25 percent more patients than beds was the norm towards the end of the period. The average length of stay was almost 3 years. Several patients stayed in the hospitals for decades or all of their lives after first admission.

### 1955-65: The period of institutionalisation and diversification of services

From the early 1950s there was a growing concern among professionals and politicians about the conditions of treatment for psychiatric patients. A government commission was established in 1951 to revise the first *mental health Act *from 1848. The commission proposed a major expansion of PNHs and PHs. Patients with a chronic disorder should not stay in the PHs for a longer period, but be transferred to a PNH when no longer in need of hospital services. This would reduce the overcrowding in the PHs. Patients in private care not receiving adequate care should be transferred to the PNHs. The commission also proposed that psychiatric departments should be established in general hospitals on the same footing as other hospital departments [30,32].

Another commission proposed improvements in the financing of mental health services, and that the counties should be responsible for them (including GHDs, PHs and PNHs) [33]. Most of the PHs run by the central government was to be transferred to the counties.

The PHs were rehabilitated and expanded. The number of beds in PHs increased by 22 percent from 1955 to 1965 (figure 5). The average number of inpatients, though, increased only marginally. The crowding in the hospitals was in this way reduced and the quality of care and treatment was raised. The occupancy rate in the hospitals declined from 125 percent in the early 1950s to 104 percent in 1965 (figure 6). The average length of stay at the PHs was reduced as well. From 1955 to 1965 it was halved (figure 3). The number of discharges was more than doubled (figure 2).

From the late 1950s, psychiatric wards were established in general hospitals (GHDs) and the number of beds in GHDs and PCs increased from 407 in 1955 to 878 in 1965 (figure 5). The average number of inpatients rose from 391 to 759. Since the average length of stay in these institutions was much shorter than in other institutions, this indicates a major increase in the total number of patients treated and that patients treated in these wards had less severe and chronic disorders.

From the early 1950s there was a major expansion of psychiatric nursing homes, built in large numbers between 1950 and 1975. The inpatient population in PNHs was tripled from 1950 to 1975 (figure 1). The number of beds and the average number of inpatients in PNHs peaked as late as 1985. In total, the numbers of beds in institutions increased by 34 percent from 1955 to 1965. The average number of inpatients was increased by 16 percent.

Expansion of the GHDs and PCs as well as shorter length of stay in the PHs indicates increased emphasis on active treatment. Still, the main focus of the psychiatric services was on long term care, and the solution to overcrowding and low standard of services was expansion of existing services and improvement in the quality of these services. Facilities for treatment and training outside the overcrowded institutions, in the communities, were still few or non-existent. However, the new 1961 *Mental Health Care Act *[34] foresaw some of these developments.

The Act replaced the law from 1848. The government aimed at opening up mental health care to new groups of patients, in new kinds of institutions, based on 'modern' principles. The indications for treatment were widened, and psychotherapy for less serious disorders, such as neuroses, was recommended. The legislation also mentioned behavioural disorders, 'bad behaviour in schools' and 'family psychiatry' [35]. These latter groups had not previously been provided mental health care. Among the new institutions advocated in the Act, the 'outpatient clinics' were to become the most important [36]. This act also stated that the state run PHs where to be transferred to the counties in which they were located, and take responsibility for prevention and psychiatric treatment and rehabilitation.

### 1965-75: Inpatient population stabilised. The onset of de-hospitalization

The first part of the period saw a marginal increase in the number of beds in psychiatric hospitals. By 1975 though, the number of beds was back at the same level as in 1965. However, the average number of inpatients was reduced throughout the period (figure 1), thereby reducing the occupancy rate from 105 to 89 percent (figure 6). The average length of stay continued to decline, see figure 3. The number of discharges grew by 48 percent, see figure 2.

The number of beds in GHDs and PCs also increased through the first part of the period, to a peak in 1971 (figure 5). The average number of inpatients displays the same pattern (figure 1). The average length of stay was only marginally reduced, from 49 to 38 days (figure 3). Still, the number of discharges increased 49 percent (figure 2)

The number of beds and the average number of inpatients in PNHs continued to grow throughout the period (figure 1 and figure 5). From the late 1960s the average length of stay in these institutions started to drop (figure 3).

The total number of beds in psychiatric institutions continued to grow, but at a much slower rate than in the previous period, and reached its peak in 1973 (figure 5). Despite the growth in bed capacity, the average number of inpatients remained on almost the same level as in 1965. It reached a peak in 1972 (figure 1). The average length of stay continued to decrease, so the number of patients treated continued to grow. By 1973 there were almost 20000 discharges, compared to 12903 in 1965 (figure 2).

A new Hospital Act was approved in 1970 [37,38]. The counties were made responsible for all specialised health services, and the financing of the institutions was improved with increased government co-payment per inpatient day.

### 1975-87: The period of de-hospitalization

In 1975 the government published a White Paper on the health policy partly as a response to the growing running costs of health institutions [39]. Norway also experienced a recession in the wake of the 1973 oil crisis. The Paper outlined the WHO principles that the health services should be based on well developed primary care services, and that treatment should be taken care of at the lowest effective level. Central government grants would replace co-payments per inpatient days. This reform came into effect in 1980. The local councils were to have formal responsibility for most primary care services. This reform came into effect in 1984.

The white paper also commented on the organisation of psychiatric services:

'Concerning the psychiatric hospitals, they should be developed so their long stay patients can be referred to psychiatric nursing homes or to their home...The differences between the mental hospitals and the psychiatric clinics and general hospital departments will fade away, aiming at developing community oriented sectorised psychiatric models...' [39]

This implied that each institution (PH, PC or GHD) should be responsible for providing most specialised psychiatric services to a geographically defined area (sector) of the county. In this way each institution would have responsibility for a smaller area, and the contact between the specialised services and the area served was supposed to improve.

From 1975 to 1987 the total number of beds in psychiatric institutions was reduced by 31 percent. The whole reduction in this period was at the PHs, were the number of beds were reduced by more than 50 percent. The average inpatient population displayed a similar pattern. Reduced average length of stay was not sufficient to compensate for reduced bed capacity, so the total number of discharges fell by eleven percent from 1977 to 1987 (figure 2).

The down-sizing of PHs then, accelerated at the end of the 1970s and in the 1980s. This also reflects an ongoing *organizational change*: The redefinition of PHs and their integration into the general hospitals. The PHs and GHDs concentrated on psychotherapy and treatment in smaller and more specialised wards. Short-term therapy became the main aim of the PHs, as it had always been for the GHDs and PCs. Taking care of long-term patients was no longer the main objective of the PHs. Long term patients were referred to PNHs which became the most frequent institutional alternative in these years. Many older patients who had stayed for years in the hospitals were transferred to nursing homes or discharged into the community.

Primary care services have traditionally been the responsibility of the local councils. During the early 1980s this responsibility was formalised and extended. From 1984 the local councils were given formal responsibility for providing most primary care services. This include health promotion and prevention of illness and injuries, school health services, health centres, child health care provided by health visitors, midwives and general practitioner services, support for families, home nursing and home help services, long term services for the elderly, and day-centres for training and work. These public welfare services are part of the 'practical assistance' that covers all kinds of help for the performance of daily-life tasks in households with persons in need of such help. This was also to include services to mental patients. From 1986 the responsibility for providing general somatic nursing homes was also transferred to the local councils. The PNHs were, however, still the responsibility of the counties.

During the late 1970s and the 1980s, specialised psychiatric services did not have first priority. Focus was on expanding primary care service, for instance for old people in nursing homes, home based care etc. In the psychiatric services focus was on giving active treatment. The counties, then, was cutting down on long time care services, while the local councils were still ill equipped to take over the responsibility.

### 1988-98: From PNHs to community based services

From the early 1980s, the role of the PNHs had been under discussion. In 1985 The Directorate of health published a report recommending that psychiatric nursing homes should be transformed to *Living and Treatment Centres*, later renamed *District Psychiatric Centres *(DPC) [40]. The DPCs should provide short-time inpatient care, day-centres and outpatient services for the local community, quite the opposite of what the PNHs had offered. Fewer patients than before had a psychiatric institution as their permanent residence. Focus for PHs and GHDs became more and more on short-term active treatment of selected patients not treated at the DPCs.

By 1991 there were 412 beds in the DPCs. In the following years they expanded rapidly, to 1492 beds in 1998 (figure 5). By then they outnumbered the PNHs (1390 beds). The number of beds in PHs, GHDs and PCs remained stable. The total number of beds was reduced by 31 percent from 1988 to 1998. The average inpatient population was correspondingly reduced. The number of discharges, however, increased by 56 percent from 1988 to 1998. In the same period, the average length of stay was reduced from 170 to 68 days.

The DPCs played a major role in increasing the number of discharges. In 1988 'other institutions' (including the DPCs) accounted for 2501 discharges, 13 percent of the total. By 1998, the DPCs alone accounted for 5738, 20 percent of the total. 'Other institutions' accounted for an additional 2345 discharges (eight percent) (figure 2).

During this period there was also a major increase in the outpatient services. The number of consultations increased from 310 000 (725 per 10000 inhabitants) in 1991 to 476 000 (1071/10000) in 1998.

### 1999-2008: The National Mental Health Program

In 1997, the government published a White Paper [41] dealing with mental health issues. The services were characterized in this way: 'Patients do not feel they get what they need; staff do not feel they do a good job, and the authorities are not able to give the population satisfactory services.' (p. 16). The *White Paper *further concluded: (a) Primary prevention is too weak. (b) In many municipalities and communities the patients do not get what they deserve; (c) there are too few beds in security wards and in psychiatric hospitals; (d) the admission thresholds for the patients are too high, making it difficult to be admitted; (e) the time span from the first symptoms of a disorder to initiated treatment is too long; (f) too many patients are discharged from the hospital too early; (g) the discharge of patients is not properly planned; (h) the aftercare or follow up after being discharged does not function properly.

The White Paper called for a major expansion and restructuring of services, both in primary care and in the specialised mental health services. The Parliament ordered the Government to present a binding plan for improved mental health services. The following year a national mental health program was approved by the parliament [42]. Over the next eight years (1999-2006, later extended to 2008) running costs for the specialised services were to expand by 29 percent (2.1 billion NOK). A similar amount was set aside for improving services provided by the local councils, for instance sheltered housing [24].

The programme called for a restructuring of the specialised services based on three pillars:

• Hospital wards should provide highly specialised services (acute wards, specialised functions)

• District Psychiatric Centres (DPCs) should provide less specialised services on a more decentralised level

• Psychiatrists and psychologists in private practice should provide services in co-operation with other mental health services.

In addition, there was to be a major expansion of primary care services provided by the local councils. The number of beds was to increase considerably in DPCs and slightly in the hospitals. Other institutions, mainly PNHs, should gradually be closed down. The total number of beds was, however, not to be reduced.

The DPCs were to play a major role, providing the following services: (a) Outpatient clinics/ambulant services, (b) Daytime treatment, (c) Short-time inpatient treatment, (d) Long term treatment and rehabilitation, (e) Consultation, supervision and support for staff in primary care services, (f) Acute services (if long distance to a PH/GHD) and crisis intervention

The number of beds in DPCs increased by 25 percent from 1998 to 2007 (figure 5). The number of beds in hospitals was, however, reduced by 13 percent. The PNHs were closed down at a much faster rate than planned and phased out by 2004. Despite the guidelines laid down in the program, the total number of beds was reduced by 21 percent from 1998 to 2007 (figure 5). The average number of inpatients was correspondingly reduced, see figure 1. The average length of stay was reduced from 66 to 31 days (figure 4). The number of discharges grew by 63 percent, see figure 2. The increase was most pronounced outside the hospitals (i.e. in DPCs and 'other institutions'). From 1998 to 2007 the average length of stay in these institutions combined was reduced from 110 to 32 days, and the number of discharges was more than doubled. During the same period the number of outpatient consultations grew from 476000 (1078/10000) to 988000 (2086/10000).

## Discussion

This section will focus on four topics: (1) General trends in the period studied. (2) De-institutionalisation, trans-institutionalisation or re- institutionalisation? (3) The reasons for the changes taking place. (4) The consequences for the patients and service providers.

### General trends 1955-2007

The total number beds and the average number of inpatients increased up to the early 1970s and have been reduced ever since.

Throughout the period there has been a continuous reduction in the average length of stay. This has been facilitated by increased capacity in GHDs and PCs from the late 1950s and the introduction of the DPCs from the late 1980s. In addition, the average length of stay has been reduced significantly in the PHs from the mid-1950s and the PNHs from the early 1970s. Despite reduced bed capacity, the number of patients treated in institutions has, with the exception of a short period during the 1980s, continuously increased. This means improved accessibility to treatment and care for the population. To characterize the period as a period of de-institutionalisation does not mean that fewer people than before is treated and cared for in the psychiatric institutions. The opposite is actually the case. The discharge rate has doubled from 1955 to 2007. Adding patients receiving out-patient treatment, the rate of inhabitants treated and nursed by the specialist mental health services increased even more. We therefore underline that de-institutionalisation in Norway during the period means reduction in the number of *beds *and in inpatient population, not in the number of *patients *treated, neither in institutions in total nor in psychiatric hospitals.

There has been a reduced threshold for being referred and admitted to the mental health services and the mental health services have become more diversified. From the 1970s out-patient clinics were founded in urban areas and about 80 District Psychiatric Centres has been established since 1990. Most patients are now treated and cared for outside the institutions, at out-patient clinics or in the municipalities. This indicates a shift in the focus of the services, from long-term care in private households and hospitals, to active treatment in smaller institutions and rehabilitation, after-care and nursing in the community.

In fact, with the exception of the 1980-87 periods, the number of patients discharged/treated has increased continuously. Adding out-patient services, the increase have been even greater.

During the whole 1950-2007 period there has been a redistribution of beds and patients. Up to the mid-1950s psychiatric hospitals and private care were the dominant forms of care. From the early 1950s until the mid-1970s there was a major expansion of the PNHs, relieving the PHs and private care of long term patients. During the 1955-65 period there were also expansion of the PHs and establishment of GHDs and PCs. The PNHs has been closed down after being the institution with the highest inpatient population in the mid-1980s The period 1950 to 2007 is therefore characterised by institutionalisation, de- institutionalisation as well as trans- institutionalisation and in the following we give an account in more detail of the redistribution of patients in different periods.

### De-institutionalisation, trans-institutionalisation or re- institutionalisation?

During the 1950-2007 the mental health services have experienced periods of institutionalisation, de-institutionalisation, trans-institutionalisation as well as re-institutionalisation. New kinds of institutions (PNH and DPC) has been established (and closed down (PNH)) and these institutions have taken over inpatients from other institutions. De-institutionalisation and trans-institutionalisation have been simultaneous processes. When analysing the redistribution of institutionalised patients in the period we also have to separate between de-institutionalisation and de-hospitalisation.

The period from the mid-1950s to the early 1970s was a period of both institutionalisation (increased number of beds and inpatient population in PHs, GHDs, PCs and PNHs), re-institutionalisation (the former private care patients were transferred to PHs and PNHs), and trans-institutionalisation (for the PHs patients referred to PNHs). It was not a period of de-institutionalisation, since the average inpatient population increased, private care excluded. With the drop in the average length of stay in PHs and the expansion of GHDs, the number of patients treated increased even more. The PNHs had taken over the PHs former role as an institution for custody and care more than treatment.

The 'de-institutionalisation' started as de-hospitalization in the early 1970s. From 1975 to 1987, the inpatient population at the PHs was halved. The average inpatient population in PNHs was stabilized and was not noticeably reduced until 15 years later; from the end of 1980s. The de-hospitalisation was at the same time a trans-institutionalisation since so-called chronic psychiatric patients were transferred from PHs to PNHs. The psychiatric nursing homes were often situated in remote rural areas and in institutions which were initially built for patients suffering from tuberculosis. Thus, what may look like de-institutionalisation in fact constitutes a trans-institutionalization and does obviously not correspond with modern ideas of mental health services.

From the early 1990s, de-institutionalisation was shifted from the PHs to the PNHs. The average inpatient population of PHs, GHDs and PCs combined has been only marginally reduced since 1990. By the end of the 1980s, the PNH was the dominant mode of care. From then on, the inpatient population of the PHNs started to decline, and by the early 2000s, most of these institutions had been closed down. Some of the PNHs were either converted into, or replaced by DPCs, providing active inpatient as well as outpatient treatment to the local community. In one sense, this might be seen as a form of trans-institutionalisation. The patients receiving inpatient treatment at these institutions, however, differs markedly from the patients in the PNH, with a patient composition more similar to the PHs/GHDs/PCs than the PNHs [43]. In another sense, then, the DPCs might be seen as the institutionalisation of new, community based services to new groups of patients.

The "rise and fall" of the psychiatric nursing homes during the period illustrates an era in the mental health services when professionals, authorities, relatives and patients believed in institutionalised long term care without treatment. Nursing could more efficiently take place in large institutions; some of them build for other patient groups. These buildings were filled with psychiatric patients in need of nursing. The rise the PNHs also illustrates trans-institutionalisation as well as a changing division of labour within the mental health services, between more active treatment at the PHs, while long term care was to be the responsibility of the PNHs. It should not be considered a shift towards de-institutionalisation and/or increased emphasis on community based services. The PNHs were organised within the same organisational structure as the PHs. These institutions played an important role in the mental health services in Norway for about 40 years. By the late 1980s, after about 30 years of existence, they had become the dominant mode of mental care, taking care of more than 50 percent of the average number of inpatients. Since then, the focus of the mental health services has turned away from long term care, and the PNHs have been closed down.

De-institutionalisation consists according to Bachrach [2] of three components, one of them are 'the development of special community-based programmes, combining psychiatric and supportive services, for the care of non institutionalised patient population.' This is not the case in Norway, at least not when we are dealing with de-hospitalisation. De-hospitalisation in Norway started as a trans-institutionalisation, from PH to PNH and at that time, in the 1970s, there were few community-based programs. Mental health services were still exclusively specialised institutional psychiatry. From the late 1980 the community based primary care in the local counties were gradually established as well as the out-patient clinics. This introduced a new era of mental health services, given priority in the National mental health program 1999 - 2008.

### Reasons for the changes taking place

Different explanations can be given for the de-institutionalisation, re-institutionalisation and trans-institutionalisation. The need for institutional care in the 1950s and 1960s may have risen due to the loss of social support for people with severe psychiatric disorders in traditional families. Urbanisation made the situation more difficult for families to take care of a patient and this was enhanced when women entered the labour market, taking professional roles instead of being domestic carers. Assistance from the public health services became necessary, and total institutionalisation was the only option offered.

The alteration of the services the last 60 years was affected by organisational changes, professional knowledge, and political-administrative intervention. In the period there have been major administrative and political reforms in the health services in general. Increased awareness and emphasis has also been placed on the patients' own preferences and requests. In the following, we will discuss the relevance of some common explanations for the de-institutionalisation of the services.

#### • The pharmacological revolution

The first anti-psychotic drugs were introduced in the early 1950s. In both the UK and the US the inpatient population in the hospitals peaked in 1954/55, and was from then on rapidly reduced [3,10,12]. It has therefore been suggested that the new drugs made living outside the hospitals possible for many patients, and thus caused the de-institutionalisation, or more precisely: de-hospitalisation. This description of the development in Britain and the United States from the mid-1950s do not fit the situation in Norway at that time. In Norway the introduction of the anti-psychotic drugs in the 1950s did not lead to an immediate or strong reduction in the number of beds or the average number of inpatients in psychiatric hospitals, nor in psychiatric institutions in general. On the contrary, the number of beds in PHs increased and the average number of inpatients was fairly stable until the late 1960s. Neither was alternative settings outside the institutions and in the communities established until the 1980s.

#### • The critique of psychiatry and the total institutions

Both inside and outside the services a view criticising the ill effects of prolonged stay within the large institutions emerged with increasing force during the 1950s [29]. Sociologists and professionals argued that the 'total institution' maintained or created dependency, passivity, exclusion and disability, causing people to be institutionalized for a long time, even for the rest of their lives [27,28,30].

Three different, yet internationally influential theoretical positions, have been widely associated with the support for de-institutionalisation, and at the same representing the anti-psychiatric movement: Erving Goffman's *Asylum *[27], Michel Foucault's *Madness and Civilisation *[44] and Thomas Szasz's *The Myth of Mental Illness *[45]. These traditions were also present in Norway, but the anti-psychiatric movement has never been very strong, and was not a main reason for downsizing the psychiatric hospitals and institutions.

#### • The expansion of the welfare state

Mechanic and Rochefort have pointed out that development of general public welfare services have influenced the de-institutionalisation of mental health services in two ways. The development of social security programs (disability pensions, public housing etc.) made it on the one hand possible for long term patients to live outside the large institutions. On the other hand, the development of the welfare state was accompanied by a critique of the standard of living in the institutions, and demands for improvement that would lead to increased costs for inpatient services [10] (see also [17]).

In Norway, a universal disability pension plan was introduced in 1959 [46], later incorporated into the *Law on Universal Social Welfare Insurance *(*Folketrygden*) [47], which was passed by the parliament in 1967. The general welfare state system, disability pensions, old age pensions, unemployment payments, public housing, and universal health insurance were a major prerequisites for the smooth process of de-institutionalization in Norway, and made it possible for people with mental disorders to be cared for outside the institutions and in their own home. Patients and their relatives also wanted more frequently to have an impact on the type and location of the services. It became more attractive, and more realistic, to provide community-based services rather than hospital based services. Whether services became cheaper to decision-makers is debatable.

#### • Cost containment and the fiscal crisis of the state

Given budgetary constraints, most governments will try to minimize, or at least contain costs. The reduction in the inpatient population from the mid-1970s coincides with the depression following the 1973 oil crisis. Cost containment was also a stated purpose of the 1975 White paper and the 1980 financial reform. The central government co-payment per inpatient days was replaced by a fixed central government grant to each county, based on the relative need for services. The economic incentives for keeping patients in institutions was in this way removed. In the 1980-87 period not only did the number of beds in PHs continue to decline, even the total number of inpatients treated per year also fell. At least in this period, de-institutionalisation might therefore be considered a means of cost reduction, or at least cost containment.

There is, however, little evidence that the costs were actually reduced. According to Statistics Norway the number of man-years in the mental services remained fairly stable from 1980-87, despite the reduced number of inpatients [48,49]. In addition, the reduction in the average number of inpatients has also continued in periods of growing resources, although on a slower pace. Since 1991, there has been a major increase in the number of man-years, despite continuous reductions in the average number of inpatients [50]. During the National mental health plan (1999-2008), the number of beds has been reduced by 20 percent, despite the stated goal that the number of beds should remain stable.

As we have seen, the average length of stay declined throughout the 1955-2007 period. In general, short stays in institutions will require more man-power per inpatient day than longer stays. At best, then, de-institutionalisation has facilitated increased focus on active inpatient and out-patient treatment, and has not been a means of cost reductions.

#### • Changing focus of services

Busfield [3] sees de-institutionalisation first and foremost as the result of a changing focus of services, from long-term care to acute treatment of patients with less serious problems. Several factors have contributed to this shift: New medical ideas undermined the support for the traditional institutions, development of alternative services made it possible to live outside of the institutions, the psychiatrist wanted to be better integrated into specialised medical services in general, and increased therapeutic optimism suggested that shorter stays were possible.

The experiences from Norway support this hypothesis. As we have seen, there has been a continuous increase in the number of patients being treated, and a continuous reduction in the number of beds and the average length of stay in institutions. Gråwe et al. [51] found a marked decrease primarily in the number of older patients in PH, GHD and PC from 1979 to 1989, that is, during the period of de-hospitalisation. Pedersen and Bjerkan [43] found a similar development both in PH/GHD/PCs and in the remaining institutions over the period 1989-2007, that is, in the period the PNHs were closed down. The 1984 primary care reform, giving the local councils formal responsibility for most primary care services, including services to people with mental problems, and closer integration of mental health services and general hospital services both support increased focus on active treatment and less emphasis on long term care.

#### • Increased emphasis on patients' rights and preferences

Over the last decades increased emphasis has been placed on patients' own views and preferences. Through the national mental health program, this has also become public policy. The programme strongly emphasis that users of services are to be involved in the planning of services, both on the individual level and on the organisational level [42].

Surveys among inpatients at the mental health institutions in Norway have revealed that many patients preferred not to stay in the PHs or in other large institutions, but to live in their own home or in a sheltered accommodation, being supported by the mental health services [52-57]. The staff at the institutions also indicated that the ideal settings for many institutionalised patients would be community care or that patients at hospitals should be transferred to other and smaller institutions. Even in 2007, approximately 30 percent of the inpatients would, according to the staff, be better off if treated and cared for in the community [58].

### Consequences of the changes taking place

Obviously, one of the main criteria for judging a health service system is to what extent it provides the population with good, efficient services. The question, then, is to what extent patients have benefited from the changes taking place over the period.

The 1955-65 period was characterised by an increase both in the number of beds both in PHs and PNHs. For patients in the hospitals this represented a reduction in the overcrowding and hopefully in the standard of living for the patients. The movement of chronic patients from large PHs to smaller, more residentially like PNHs had probably the same effect. The move of patients from private care to PNHs might also have increased the patients' standard of living. At least, that was the stated goal.

The de-institutionalisation of services gained momentum from the mid-1970, with the down-sizing of the PHs. Several surveys of in-patients conducted since the late 1970s have indicated that many patients would prefer services in the community rather than in the institutions. Likewise, according to staff, many patients would be better off; receiving community based services rather than staying on in a psychiatric institution.

The question, then, is to what extent community based services became available. There are clear indications that this was not the case during the first 10-20 years of de-institutionalisation. Long-term patients were discharged before alternative community based services were available. This problem was accentuated by institutional barriers. The counties were responsible for the psychiatric institutions; while the local councils were responsible for most community based services.

This was one of the main motives behind The National Mental Health Program. There was a need for the planning of services based on patients' needs, not administrative and institutional boundaries, as well as better integration of services. In order to achieve this, the central government provided ear-marked grants. To get these grants, counties and local councils had to make plans for the development of services, and these plans had to be approved by government agencies.

Evaluation studies from recent years have documented that the recent alterations, especially the National Programme initiated in 1998, has had a positive impact on access and equity, quality and efficacy, fairness, patients rights, protection, participation and treatment outcome [24,43,50,59].

The closing down of the large institutions, build for custody more than treatment, has been to the benefit of the institutionalized patients and the priority given to outpatient treatment has been an advantages to patients able to live in their own home. The services in Norway are characterized bye more diversity throughout the whole period. This also means that the treatment and care has to a larger extent become more tailored for every person in need of treatment or care.

The many smaller institutions established around in the local communities and with responsibility for a target area, the DPSs, with some beds and with outpatient service, are more in accordance with the patients' preferences and also with what psychiatrists and psychologists think is appropriate. It has also improved the accessibility to the services for everybody.

The *Act of Patients' Rights *from 2001 and the establishment of a Patient *Ombudsman *has improved patients' privileges and legal rights in general, for instance the right of access to the medical records and to chose where to be treated. Any person, a patient or a relative, may contact the Patient Ombudsman and request that a case be taken up for consideration.

In a recent comparison of USA, Great Britain, Canada and Norway, Norway was unique among the four countries in its vision of integrated mental health services grounded with equal accessibility for everybody [60]. It is said in this international comparison of mental health systems that other countries can learn from Norway. The Norwegians adopted centralized financing and administration of mental health services to produce a more standardized and equitable system for delivering high quality care.

Another lesson from Norway pertains to the vital role of workforce planning [24] without redistribution of personnel according to population density and prevalence rates, it is unlikely that a national policy authorizing universal access will be fully implemented or that the needs for mental health services will be met equitably, and especially in rural areas.

## Summary and conclusions

We have in this Paper traced changes in the inpatient care of Norwegian mental health services over the period 1950-2007. Six distinct periods can be separated out:

• Before 1955: The asylum period, characterised by long term care in overcrowded psychiatric hospitals and in private care.

• 1955-65: Institutionalisation and trans-institutionalisation. A major expansion of PNHs and GHDs took place in this period, as well as improved standard of living in the PH. Private care was gradually reduced. The average number of patients in institutions increased. The average length of stay in PH is more than halved. The total number of discharges from PHs was more than doubled.

• 1965-75 Inpatient population is stabilized. De-hospitalisation sets in at the end of the period. The average length stay continues to go down, both in PHs, PC/GHD, and from the 1970s, also in PNHs. Private care continues to be reduced.

• 1975-87: De-hospitalisation. The average number of inpatient in PHs is more than halved. At the end of the period, the PNHs are the dominant mode of care. Despite reduced length of stay in all institutions, the total number of discharges fell from 1980 to 1987.

• 1988-98: From PNHs to DPCs. This period is characterized by a steady inpatient population at the PHs, PCs, and GHD. De-institutionalisation is shifted to the PNHs. By the early 2000s, most of them had been closed down. The PNHs are replaced by DPCs delivering services to the local community and increasing its share of inpatients.

• 1999-2008: The National Mental Health Program. The DPCs are expanded. The remaining PNHs are closed down. The total average number of patients is reduced by approximately 20 percent, while the number of discharges increases by 63 percent.

Contrary to the experiences in the UK and the US, the pharmacological model does not fit the Norwegian experiences, since de-institutionalisation started at a much later date. The critique of psychiatry and the total institutions fits better with the time period, but the anti-psychiatric movement was never very strong in Norway. The welfare state made living outside institutions possible, and might have facilitated de-institutionalisation. Cost containment might have played a part in the downsizing of the PHs in 1980-87 period. However, de-institutionalisation has also continued in periods with increasing resources. Changing focus of services, from long term care to short time active treatment seems the most plausible explanation. Increased focus on patients' preferences has also supported the structural changes.

## Tables

Table 1, Table 2 and Table 3 give population based ratios for beds, average inpatient population and discharges by year and institutional setting.

**Table 1:** Average inpatient population by institutional setting, mental health services in Norway 1950-2007- ratios per 10,000 inhabitants

Year	PHs	PCs and GHDs	PHs, PCs and GHDs	Other institutions	DPCs	Sum, all institutions	Private care	Sum all patients
1950	24.1	0.9	25.0	4.1		29.1	22.6	51.7
1951	24.1	0.9	24.9	4.5		29.5	22.2	51.6
1952	24.2	0.8	25.0	4.9		30.0	21.4	51.3
1953	24.2	0.8	25.0	5.3		30.3	20.5	50.8
1954	24.1	1.0	25.1	5.7		30.8	20.0	50.8
1955	24.1	1.1	25.2	5.9		31.1	19.6	50.7
1956	23.8	1.1	25.0	6.7		31.6	18.4	50.1
1957	23.6	1.3	25.0	6.8		31.7	17.2	48.9
1958	23.7	1.6	25.3	6.9		32.2	16.9	49.2
1959	23.8	1.7	25.5	8.0		33.5	16.6	50.1
1960	23.2	1.7	25.0	8.5		33.5	16.0	49.5
1961	22.8	2.3	25.1	8.6		33.8	15.7	49.4
1962	22.8	2.3	25.1	9.1		34.2	15.0	49.2
1963	22.5	2.0	24.5	8.9		33.4	14.7	48.1
1964	22.5	2.1	24.7	9.2		33.8	13.9	47.7
1965	22.8	2.0	24.8	8.5		33.3	13.0	46.3
1966	22.4	2.1	24.5	8.3		32.8	12.6	45.4
1967	22.2	2.3	24.5	8.1		32.7	11.5	44.2
1968	21.8	2.5	24.3	8.4		32.7	10.9	43.7
1969	21.4	2.8	24.1	8.9		33.0	10.8	43.7
1970	20.7	2.9	23.6	8.5		32.0	10.0	42.0
1971	20.3	3.0	23.4	9.4		32.8	8.7	41.5
1972	19.8	2.8	22.6	10.0		32.6	8.6	41.2
1973	19.3	2.8	22.0	10.1		32.1	8.1	40.2
1974	18.7	2.4	21.1	10.2		31.3	7.3	38.6
1975	18.0	2.2	20.1	10.8		30.9	6.6	37.5
1976	17.6	2.3	19.9	10.9		30.7	6.2	37.0
1977	16.3	2.1	18.5	10.8		29.2	5.4	34.6
1978	15.5	1.9	17.4	10.7		28.2	5.1	33.3
1979	15.1	1.9	16.9	10.7		27.6	4.6	32.2
1980	13.5	1.9	15.4	10.5		25.9	4.2	30.1
1981	12.1	1.9	14.0	10.5		24.5	3.8	28.3
1982	10.9	1.8	12.7	10.5		23.2	3.5	26.7
1983	10.0	1.7	11.7	10.5		22.2	3.1	25.4
1984	9.1	1.7	10.9	10.6		21.5	2.6	24.1
1985	8.6	1.6	10.3	10.4		20.6	2.4	23.0
1986	8.1	1.7	9.8	10.4		20.2	2.2	22.4
1987	7.4	1.6	9.0	10.4		19.5	2.0	21.5
1988	6.6	1.7	8.3	10.1		18.4	1.8	20.2
1989	6.0	1.6	7.6	9.8		17.3	1.6	19.0
1990	5.5	1.7	7.2	9.0		16.2	1.5	17.7
1991			6.6	7.6	0.8	15.0	1.3	16.3
1992			6.6	7.0	1.1	14.7	1.2	15.9
1993			6.5	6.1	1.7	14.2	1.1	15.3
1994			6.2	5.0	2.0	13.3	1.0	14.2
1995			6.1	4.7	2.0	12.9	0.9	13.8
1996			6.1	4.5	2.0	12.7		
1997			6.3	3.7	2.4	12.4		
1998			6.4	2.8	2.7	11.9		
1999			6.4	2.3	2.9	11.7		
2000			6.2	2.1	2.9	11.2		
2001			6.0	1.6	3.1	10.7		
2002			5.8	1.2	3.4	10.4		
2003			5.7	1.1	3.2	10.0		
2004			5.4	0.5	3.7	9.6		
2005			5.3	0.4	3.6	9.3		
2006			5.5	0.3	3.3	9.1		
2007			5.1	0.3	3.2	8.6		

**Table 2:** Beds by institutional setting, mental health services in Norway 1950-2007- Ratios per 10 000 inhabitants

Year	PHs	PCs and GHDs	PHs, PCs and GHDs	Other institutions	DPCs	Sum all institutions	Private care	Sum all patients
1950	19.3	0.9	20.2	4.1		24.3	22.6	46.8
1951	19.4	0.9	20.3	4.5		24.9	22.2	47.0
1952	19.4	0.9	20.3	4.9		25.3	21.4	46.6
1953	19.5	0.9	20.4	5.3		25.7	20.5	46.2
1954	19.4	1.0	20.4	5.9		26.3	20.0	46.3
1955	19.3	1.2	20.5	6.0		26.5	19.6	46.1
1956	19.4	1.2	20.5	6.8		27.4	18.4	45.8
1957	19.4	1.4	20.8	6.8		27.5	17.2	44.7
1958	20.2	1.7	21.9	7.0		28.9	16.9	45.9
1959	20.6	1.8	22.3	8.1		30.4	16.6	47.0
1960	20.1	1.8	21.9	8.5		30.4	16.0	46.4
1961	20.5	2.3	22.8	8.7		31.5	15.7	47.2
1962	21.4	2.4	23.8	8.9		32.7	15.0	47.7
1963	21.7	2.3	24.0	8.9		32.8	14.7	47.6
1964	22.0	2.4	24.4	9.1		33.5	13.9	47.3
1965	21.8	2.3	24.2	8.5		32.7	13.0	45.7
1966	21.4	2.3	23.7	8.3		31.9	12.6	44.6
1967	21.5	2.7	24.2	8.0		32.1	11.5	43.7
1968	21.4	2.8	24.2	8.3		32.5	10.9	43.5
1969	21.5	3.1	24.6	9.1		33.7	10.8	44.5
1970	21.6	3.4	25.0	8.8		33.9	10.0	43.9
1971	21.3	3.4	24.7	9.5		34.3	8.7	43.0
1972	21.1	3.3	24.4	10.0		34.4	8.6	43.0
1973	21.2	3.2	24.4	10.2		34.6	8.1	42.7
1974	20.7	2.8	23.5	10.3		33.8	7.3	41.1
1975	20.2	2.6	22.8	10.9		33.7	6.6	40.3
1976	19.4	2.8	22.2	11.1		33.3	6.2	39.5
1977	18.9	2.6	21.5	11.1		32.6	5.4	38.0
1978	18.3	2.5	20.8	11.0		31.8	5.1	36.9
1979	17.3	2.4	19.7	10.9		30.7	4.6	35.3
1980	15.9	2.5	18.3	10.9		29.2	4.2	33.4
1981	15.3	2.5	17.8	11.1		28.8	3.8	32.7
1982	13.6	2.3	15.9	11.3		27.2	3.5	30.7
1983	12.4	2.2	14.6	11.4		26.0	3.1	29.1
1984	10.9	2.1	12.9	11.6		24.5	2.6	27.1
1985	9.8	2.1	11.9	11.2		23.1	2.4	25.5
1986	9.3	2.2	11.5	11.3		22.8	2.2	25.0
1987	8.9	2.0	11.0	11.3		22.3	2.0	24.3
1988	7.6	2.0	9.6	10.9		20.5	1.8	22.3
1989	6.6	2.0	8.6	10.6		19.2	1.6	20.8
1990	6.3	2.0	8.2	9.9		18.1	1.5	19.6
1991			7.4	8.4	1.0	16.8	1.3	18.1
1992			7.3	7.8	1.4	16.4	1.2	17.6
1993			7.1	6.7	2.0	15.8	1.1	16.9
1994			6.9	5.7	2.5	15.0	1.0	16.0
1995			6.8	5.5	2.4	14.6	0.9	15.5
1996			6.8	5.2	2.3	14.2		
1997			6.8	4.1	2.9	13.8		
1998			7.0	3.1	3.4	13.5		
1999			6.9	2.6	3.5	13.0		
2000			6.7	2.5	3.6	12.8		
2001			6.5	1.8	3.9	12.2		
2002			6.6	1.4	4.1	12.0		
2003			6.4	1.2	3.9	11.6		
2004			6.1	0.6	4.5	11.2		
2005			5.9	0.5	4.4	10.8		
2006			6.0	0.4	4.1	10.6		
2007			5.7	0.4	3.9	10.1		

**Table 3:** Discharges by institutional setting, mental health services in Norway 1950-2007- ratios per 10,000 inhabitants

Year	PHs	PCs and GHDs	PHs, PCs and GHDs	Other institutions	DPCs	Sum all institutions
1950	8.5			0.0		
1951	8.5			0.0		
1952	8.4			0.0		
1953	8.6			0.0		
1954	8.5			0.8		
1955	8.8			0.9		
1956	9.4			1.1		
1957	10.0			1.1		
1958	10.3			1.1		
1959	13.3			1.3		
1960	13.3			1.4		
1961	14.0			1.6		
1962	16.4			1.5		
1963	17.7			1.5		
1964	18.3	15.9	34.2	1.5		35.8
1965	18.1	15.0	33.1	1.4		34.5
1966	18.4	16.2	34.5	1.4		35.9
1967	18.3	18.2	36.4	1.3		37.8
1968	18.8	20.0	38.8	1.5		40.3
1969	19.3	21.7	40.9	1.5		42.4
1970	20.4	23.4	43.8	1.5		45.3
1971	23.0	24.2	47.2	1.7		48.8
1972	23.4	23.6	47.0	2.3		49.3
1973	24.3	23.7	48.0	2.3		50.3
1974	24.5	21.8	46.3	2.3		48.6
1975	24.8	20.7	45.5	2.6		48.1
1976	26.6	22.3	48.9	2.5		51.4
1977	26.5	21.1	47.5	2.4		49.9
1978	27.4	18.9	46.2	2.2		48.5
1979	28.3	19.6	47.9	2.8		50.7
1980	26.4	19.5	45.8	2.9		48.7
1981	22.8	19.5	42.3	2.8		45.1
1982	23.3	18.6	42.0	3.2		45.2
1983	24.0	20.4	44.4	3.5		47.9
1984	24.3	18.7	43.0	4.1		47.1
1985	22.4	17.2	39.6	3.8		43.4
1986	21.3	16.8	38.2	4.8		43.0
1987	20.6	16.5	37.1	5.9		43.0
1988	21.8	16.7	38.5	5.9		44.4
1989	22.1	17.6	39.7	6.7		46.4
1990	21.2	18.8	40.0	7.6		47.6
1991			38.7	6.5	2.0	47.3
1992			40.4	6.6	3.0	50.0
1993			40.9	6.5	4.0	51.4
1994			40.6	7.0	5.2	52.8
1995			41.8	8.0	5.1	55.0
1996			43.6	8.6	7.3	59.5
1997			46.1	6.8	11.1	64.0
1998			47.5	5.3	12.9	65.7
1999			50.7	4.6	12.8	68.1
2000			52.0	5.1	16.0	73.1
2001			52.1	5.0	18.5	75.7
2002			55.0	3.6	23.1	81.7
2003			58.7	3.6	25.8	88.2
2004			56.6	1.3	31.6	89.5
2005			58.2	1.0	34.5	93.8
2006			63.5	0.8	37.3	101.6
2007			60.5	0.6	39.5	100.7

## Competing interests

The authors declare that they have no competing interests.

## Authors' contributions

PBP collected the data, and wrote the material and methods section. PBP is also the main author of the results section, with additional comments supplied by AK. Both authors have contributed to the background and to the discussion sections. Both authors read and approved the final manuscript.
